# Neuropsychiatric Burden of SARS-CoV-2: A Review of Its Physiopathology, Underlying Mechanisms, and Management Strategies

**DOI:** 10.3390/v16121811

**Published:** 2024-11-21

**Authors:** Aliteia-Maria Pacnejer, Anca Butuca, Carmen Maximiliana Dobrea, Anca Maria Arseniu, Adina Frum, Felicia Gabriela Gligor, Rares Arseniu, Razvan Constantin Vonica, Andreea Loredana Vonica-Tincu, Cristian Oancea, Cristina Mogosan, Ioana Rada Popa Ilie, Claudiu Morgovan, Cristina Adriana Dehelean

**Affiliations:** 1 Department of Toxicology, Drug Industry, Management and Legislation, Faculty of Pharmacy, “Victor Babeş” University of Medicine and Pharmacy, 2nd Eftimie Murgu Sq., 300041 Timişoara, Romania; aliteia.pacnejer@ulbsibiu.ro (A.-M.P.); cadehelean@umft.ro (C.A.D.); 2 Preclinical Department, Faculty of Medicine, “Lucian Blaga” University of Sibiu, 550169 Sibiu, Romania; carmen.dobrea@ulbsibiu.ro (C.M.D.); anca.arseniu@ulbsibiu.ro (A.M.A.); adina.frum@ulbsibiu.ro (A.F.); felicia.gligor@ulbsibiu.ro (F.G.G.); razvanconstantin.vonica@ulbsibiu.ro (R.C.V.); loredana.vonica@ulbsibiu.ro (A.L.V.-T.); claudiu.morgovan@ulbsibiu.ro (C.M.); 3 County Emergency Clinical Hospital “Pius Brînzeu”, 300723 Timișoara, Romania; arseniurares@gmail.com; 4 Department of Pulmonology, Center for Research and Innovation in Personalized Medicine of Respiratory Diseases, “Victor Babeş” University of Medicine and Pharmacy, 300041 Timișoara, Romania; oancea@umft.ro; 5 Department of Pharmacology, Physiology and Pathophysiology, Faculty of Pharmacy, “Iuliu Haţieganu” University of Medicine and Pharmacy, 400029 Cluj-Napoca, Romania; cmogosan@umfcluj.ro; 6 Department of Endocrinology, Faculty of Medicine, “Iuliu Haţieganu” University of Medicine and Pharmacy, 3-5 Louis Pasteur Street, 400349 Cluj-Napoca, Romania; ioana.ilie@umfcluj.ro; 7 Research Center for Pharmaco-Toxicological Evaluations, Faculty of Pharmacy, “Victor Babeş” University of Medicine and Pharmacy, Eftimie Murgu Square No. 2, 300041 Timişoara, Romania

**Keywords:** SARS-CoV-2, neuropsychiatric manifestations, COVID-19, neuroinflammation, pharmacotherapy, biomarkers, long COVID-19

## Abstract

The COVID-19 outbreak, caused by the SARS-CoV-2 virus, was linked to significant neurological and psychiatric manifestations. This review examines the physiopathological mechanisms underlying these neuropsychiatric outcomes and discusses current management strategies. Primarily a respiratory disease, COVID-19 frequently leads to neurological issues, including cephalalgia and migraines, loss of sensory perception, cerebrovascular accidents, and neurological impairment such as encephalopathy. Lasting neuropsychological effects have also been recorded in individuals following SARS-CoV-2 infection. These include anxiety, depression, and cognitive dysfunction, suggesting a lasting impact on mental health. The neuroinvasive potential of the virus, inflammatory responses, and the role of angiotensin-converting enzyme 2 (ACE2) in neuroinflammation are critical factors in neuropsychiatric COVID-19 manifestations. In addition, the review highlights the importance of monitoring biomarkers to assess Central Nervous System (CNS) involvement. Management strategies for these neuropsychiatric conditions include supportive therapy, antiepileptic drugs, antithrombotic therapy, and psychotropic drugs, emphasizing the need for a multidisciplinary approach. Understanding the long-term neuropsychiatric implications of COVID-19 is essential for developing effective treatment protocols and improving patient outcomes.

## 1. Introduction

In late 2019, several cases of an unknown form of pneumonia emerged in Wuhan, China, rapidly spreading across Asia and then all over the globe. The outbreak was ultimately confirmed to be caused by a new type of coronavirus [1,2,3]. It caused symptoms similar to those of the severe acute respiratory syndrome (SARS) in 2003, caused by severe acute respiratory syndrome coronavirus 1 (SARS-CoV-1) [4]. The severe acute respiratory syndrome coronavirus 2 (SARS-CoV-2) virion has four structural proteins: the S (spike) glycoprotein, responsible for the spikes of the virus; the envelope (E) protein; the membrane (M) protein; and a nucleocapsid with helical symmetry (N) [5]. In vivo, SARS-CoV-2 interacts with the angiotensin-converting enzyme 2 (ACE2) via the outer membrane “S” protein. Peripheral ACE2 contributes to angiotensin II conversion, impacting blood pressure regulation, while in the central nervous system, ACE2 plays multiple roles in brain injury recovery, stress response, and memory function [6,7]. The SARS-CoV-2 genome is a single-stranded ribonucleic acid (RNA) molecule, which makes the virus more susceptible to mutation and rapid adaptation, allowing it to spread from one species to another [8,9,10,11]. The genome sequence of SARS-CoV-2 can cause the dysregulation of cytokine activity, i.e., an alteration of the immune response [11,12]. Also, several studies have shown that patients infected with SARS-CoV-2 develop hyperinflammatory syndrome associated with increased circulating cytokine levels, like TNFα and IL-6.

In February 2020, WHO named the SARS-CoV-2-induced infection as the “new Coronavirus Disease 2019” (COVID-19) [13]. Although COVID-19 is primarily classified as an acute respiratory syndrome, it may also cause dysfunction of several organs and systems, particularly the central and peripheral nervous systems, given the virus’s ability to impact multiple organs. This happens particularly in patients who develop severe forms of the disease, where an excessive inflammatory cascade can be observed, disrupting the function of vital organs (cytokine storm), with subsequent bleeding disorder, low oxygen levels, liver impairment, septicemia, and acute kidney impairment (AKI). As far as neurological manifestations are concerned, these are often seen in COVID-19 patients, and range from light and non-specific symptoms, like headache, dizziness, fatigue, myalgia, anosmia, and ageusia, to more serious events such as stroke, delirium, coma, and encephalopathy [14,15,16,17,18,19,20,21,22,23]. Further studies show that a considerable number of COVID-19 patients face persistent neuropsychiatric alterations that can lead to mood changes like depression, anxiety, post-traumatic stress disorder (PTSD), and decreased cognitive function [24,25,26,27,28].

Various neurological symptoms can represent initial signs of COVID-19 and can develop in patients with or without underlying disease [29,30,31].

The development of neurological symptoms during hospitalization in patients with severe SARS-CoV-2 infection, as well as a history of neurological conditions, are associated with a higher mortality in COVID-19 disease [32,33,34,35]. Pre-existent metabolic syndromes, older age, and a dysregulated immune response are also key risk factors for increased severity and mortality due to COVID-19 infection [23]. The neuropsychiatric effects of SARS-CoV-2, like depression and cognitive impairment, highlight the need for comprehensive care, as similar inflammatory and lifestyle-related mechanisms are seen in conditions such as ischemic heart disease. For example, inflammatory responses and stressors associated with COVID-19 disease can worsen mood disorders and cognitive function, just as depression exacerbates outcomes in ischemic heart disease patients. This parallel suggests that COVID-19 patients, especially those experiencing both neuropsychiatric and systemic symptoms, could benefit from management strategies that address both mental health and physical well-being [36,37]. However, the pathways leading to such neuropsychiatric alterations in COVID-19 and their long-term consequences are still a topic for debate among researchers [38,39,40].

Different studies suggested a higher negative impact on mental health during COVID-19 in the low- and middle-income countries compared to high-income countries. Often, poor mental health support facilities represent the main cause of the increased incidence of neurologic and psychiatric disorders (e.g., depression, anxiety, neurocognitive disorders, etc.) [41,42,43].

However, it was not only the infection that caused damage, but lockdown had a negative impact on mental health during pandemic, too. Thus, a higher number of cases of psychiatric disorders as depression, anxiety, insomnia, etc., were reported. Also, ADHD was reported more frequently in children because of school closures. Additionally, burnout was frequently reported in healthcare professionals during this pandemic [44].

## 2. Classification and Underlying Factors of COVID-19 Disease

Neurological and neuropsychiatric involvement has been widely reported with COVID-19 and can occur both in the acute phase of the disease and in the post-infection recovery period. According to National Institute for Health and Care Excellence (NICE), COVID-19 can be classified into three distinct phases, starting from the time of infection and the onset of symptoms:

1. acute phase of COVID-19 infection: first 4 weeks after disease onset [45,46];
2. subacute phase of COVID-19: this includes manifestations occurring between weeks 4 and 12 after the onset of the acute phase [46,47];
3. chronic phase of COVID-19: this category includes symptoms that last for more than 12 weeks after the acute phase onset, and which are not consistent with a different diagnosis [48,49].

Post-COVID-19 syndrome is an umbrella term for symptoms, signs, and conditions persisting or appearing 4 weeks after the acute phase of infection. Patients experience persistent symptomatology following the SARS-CoV-2 infection that cannot be related to any other disease [46,49]. Post-COVID-19 syndrome is a complex condition with symptoms and mechanisms that have yet to be fully understood. Some studies suggest that high levels of D-dimer, C-reactive protein, and the presence of lymphopenia may be associated with an increased susceptibility to develop post-COVID-19 symptoms [50,51,52,53]. An abundance of studies has been identified in the literature which mention neurological symptoms of post-COVID-19.

One of the most important determinants of the neurological and psychiatric outcomes in COVID-19 patients is host immunity. The direct cytopathic effects of SARS-CoV-2 can lead to significant neurological damage and dysfunction, manifesting as a range of neurological sequelae. These sequelae are often exacerbated by neuroinflammation, which contributes to neurological and psychiatric symptoms.

Another key factor is the expression of ACE2 and other receptors. Systemic inflammation and the cytokine storm induced by SARS-CoV-2 infection can cause endothelial dysfunction and vasculopathy [54]. Dysregulation of the renin–angiotensin–aldosterone system (RAAS) may also contribute to neurological manifestations. The SARS-CoV-2 virus enters host cells by binding to the S protein and ACE2. This results in endothelial inflammation, endothelial and mitochondrial dysfunction, and inactivation of endothelial nitric oxide synthetase (eNOS), the enzyme responsible for nitric oxide (NO) production [55]. This can lead to the deregulation of the renin–angiotensin and kinin–kinase systems, affecting both cardiovascular and cerebrovascular balance [56].

The endothelium dysfunction produced by SARS-CoV-2 infection can aggravate adverse events such as inflammation and microvascular thrombosis in severe cases, including pulmonary thrombosis complications [57].

Viral factors, such as mutations and variants of the SARS-CoV-2 virus, play an important role too. These factors can lead to coagulopathy and thrombosis, which are critical pathological processes underlying many neurological complications [58]. These complications include the same wide range of neurological symptoms seen in neuroinflammation and RAAS dysregulation.

Other factors include pre-existing neurodegenerative diseases, like dementia or Parkinson’s disease, where the SARS-CoV-2 infection may exacerbate the underlying disease and lead to more severe outcomes, such as cerebrovascular events, due to thrombotic microangiopathy and hypercoagulable state, and anxiety [59,60].

**Figure 1:**
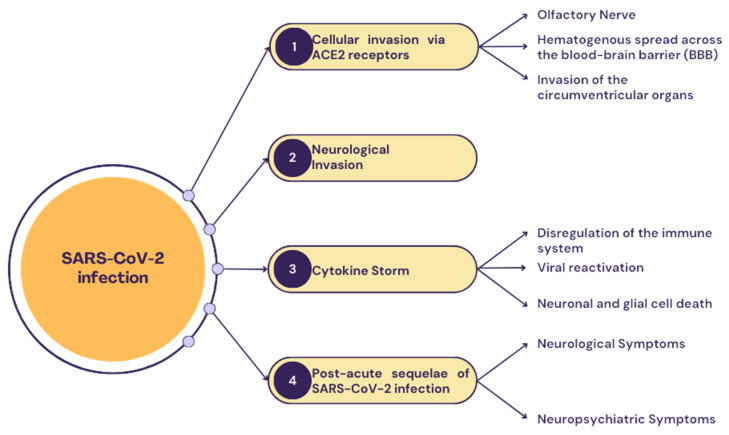
Proposed pathways of neuropsychiatric manifestations in SARS-CoV-2 infection.

The main proposed pathways by which SARS-CoV-2 infection may lead to neuropsychiatric manifestations, are summarized in Figure 1.

## 3. Physiopathology of Neuropsychiatric Presentations Associated with COVID-19 Disease

The pathophysiology of COVID-19 involves complex interactions between oxidative stress and inflammation, with viral binding to ACE2 initiating endothelial and mitochondrial dysfunction that exacerbates the production of reactive oxygen species, further promoting a pro-inflammatory state and endothelial damage. The cytokine storm observed in severe cases of COVID-19 leads to a cascade of systemic inflammation, endotheliosis, and a prothrombotic environment, highlighting the role of oxidative stress and inflammation in the neurological and vascular complications associated with the disease [61].

**Figure 2:**
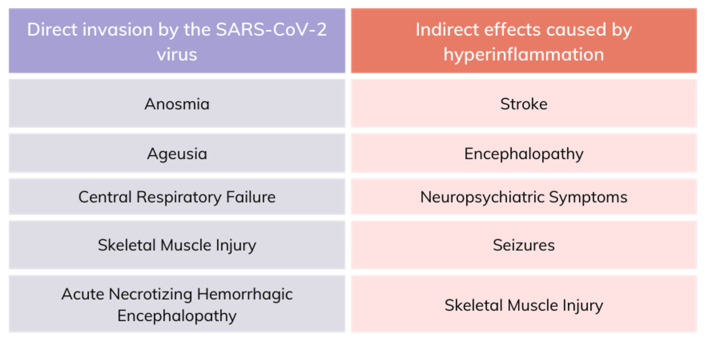
The impact of the SARS-CoV-2 infection on the nervous system and the resulting injuries.

The neurological alterations induced by COVID-19 disease are the result of complex pathogenic mechanisms. These include viral neuroinvasion, causing direct neuronal damage, cerebrovascular hypoxia, ischemia, increased inflammation, and increased coagulability. Typical neurological findings indicate damage to the nervous system, both central and peripheral, the most seen symptoms being dizziness, headache, hyposmia, and dysgeusia. Figure 2 presents the impact of the SARS-CoV-2 infection on the nervous system and its associated manifestations.

### 3.1. Experimental Studies

Animal studies have provided valuable insights into the mechanisms by which SARS-CoV-2 may affect the CNS and contribute to neurological symptoms. Experiments on mice, particularly those genetically modified to overexpress ACE2 receptors, allow researchers to observe the virus’s ability to invade the brain, disrupt the BBB, and impact various neural cells. These studies are crucial for understanding the virus’s neurotropism, the immune response within the CNS, and potential neurodegenerative effects, offering important knowledge that can complement clinical findings in human studies.

Experiments in which the SARS-CoV-2 virus was administered intranasally to ACE2 overexpressing mice were able to demonstrate the neurotropism of the virus in the brain [62]. Furthermore, the spike protein of the virus crossed the blood–brain barrier (BBB) and reached the parenchyma by adsorptive transcytosis, a vesicle-dependent transport mechanism [63]. Also, microglia have been found to play a role in the process of synapse elimination that leads to memory deficits following a viral infection [64,65].

### 3.2. Clinical Studies

The literature describes various pathways for SARS-CoV-2 penetration into the Central Nervous System (CNS), in particular, retrograde transport through peripheral nerves and viruses entering the brain through the olfactory pathway that supposed the invasion of the virus in the nasal neuroepithelium, the olfactory bulb, and its cortical projections [66,67,68,69]. After its binding by the olfactory nerve terminals, the virus is internalized by endocytosis. Then, it is transported to different brain regions through the circulatory system crossing the blood–brain barrier (BBB) [70,71].

This can be achieved either through the endothelial cells, through the migration of infected leukocytes, or by reaching the cerebrospinal fluid (CSF) through the epithelial cells of the choroid plexus [70].

**Figure 3:**
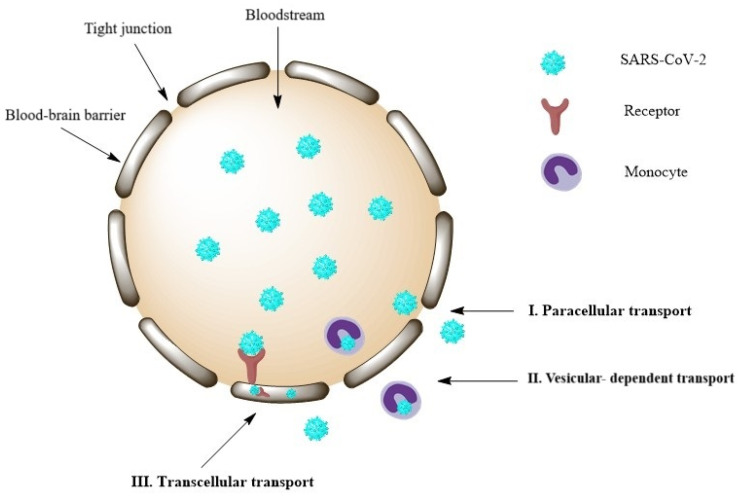
Proposed underlying mechanisms for the neurological aspects of COVID-19 disease [75].

Experiments in which SARS-CoV-2 was administered intranasally to ACE2 overexpressing mice were able to demonstrate the neurotropism of the virus in the brain [62]. In mice, the spike protein of the virus crossed the blood–brain barrier (BBB) and reached the parenchyma by adsorptive transcytosis, a vesicle-dependent transport mechanism (Figure 3) [63].

Certain studies suggest that SARS-CoV-2 may be responsible for infecting neurons as well as astrocytes, therefore leading to neurodegeneration. Furthermore, extensive protein expression and infectious viral fragments were found in SARS-CoV-2-infected neutrospheres and brain organoids. This suggests that SARS-CoV-2 can not only infect these cells, but also replicate within them, potentially leading to cell death and loss of synapses in neurons. Most studies in the literature also indicate that brain infiltration by the SARS-CoV-2 virus or its viral proteins might, potentially, cause neurological deficits in infected individuals, although it is known that coronaviruses are not primarily neurotropic viruses [25,62,72,73,74].

Viral infections of the brain can cause temporary or long-term neurological or psychiatric dysfunction, like anxiety or depression, and motor deficits. Furthermore, inflammation in the brain and an abnormal inflammatory response can disrupt neuron function and cause long-term deficits, resulting in impaired synapses and memory [64,65,76,77].

Microglia cells are a type of glial cell found throughout the brain, spinal cord, the retina, and the olfactory bulb. They serve as the primary and most crucial line of active immune defense in the CNS and are essential for normal CNS function, during both development and response to injury. They also regulate different inflammation responses, including repair, cytotoxicity, regeneration, and immunosuppression, through different activation states or phenotypes [78,79,80,81,82].

In the context of neurodegeneration, microglia are involved in both protective and potentially harmful processes because they can release proinflammatory cytokines, which can contribute to the progression of neurodegenerative disease, but they can also help to clear neuronal debris and damaged neurons, which is beneficial. Dysregulation of microglial function has been implicated in several neurodegenerative diseases, including Alzheimer’s disease, Parkinson’s disease, and multiple sclerosis [76,77,79,83]. Also, the process called “microglia priming” is the process by which microglial cells change their morphology and become a primary source of inflammatory cytokines [84]. It is known that microglia and macrophages express ACE2 but most blood immune cells do not, suggesting other receptors like toll-like receptors (TLRs) may be involved in the inflammatory response in COVID-19 disease [85,86,87].

Another type of glial cell that contributes to the development of neurological injury and age-related cognitive decline is the astrocytes, which have been shown to be incredibly diverse in their functions. This heterogeneity means that astrocytes play different roles in brain health and pathology, and their functions may change as the brain ages [88]. Astrocytes are involved in processes such as supporting neuronal function, contributing to the integrity of the BBB, regulating blood flow, neurotransmitter regulation, neuronal repair and scarring, and are responsible for producing neurotrophic factors and anti-inflammatory cytokines, like interleukin (IL)-10 [89,90,91,92]. On the other hand, reactive astrocytes are astrocytes that morphologically, molecularly, and functionally remodel in response to CNS injury or infection, when certain proinflammatory factors are released in the microglia, specifically complement component 1q (C1q), IL-1α and tumor necrosis factor alpha (TNFα). These factors elicit the functional change of astrocytes into A1 reactive astrocytes, which are harmful and release a toxic factor which kills neurons and oligodendrocytes. This remodeling has been known for over a century, but there is still uncertainty and controversy regarding the role of reactive astrocytes in CNS disorders, recovery, and senescence [93,94,95,96,97,98,99,100].

SARS-CoV-2 infection has been shown in several brain autopsy studies to be responsible for a local inflammatory response, microglial activation, signs of hypoxia, cerebral infarcts, and infection of astrocytes, which may lead to impaired neuronal viability and therefore explain some of the neurological symptoms associated with COVID-19, such as fatigue, depression, and “brain fog” [25,62,101,102,103,104]. Astrocyte activation, along with increased levels of biomarkers of CNS injury such as neurofilament light chain (NfL), glial fibrillary acidic protein (GFAP) and total tau protein, were also observed in the CSF of patients with COVID-19 and were correlated with disease severity and duration of intensive care [105,106,107,108].

The CNS has unique immune responses compared to peripheral tissues due to its specialized structure and function and the BBB plays a crucial role in shaping these responses [109]. As a response to the infection, proinflammatory cytokines are released and several studies suggest that TNFα, IL-1β, and IL-6 are significantly increased during infections and can cause learning and memory impairments. Also, TNF and IL-1β are incriminated to disrupt the BBB, while IL-1β is linked to the degeneration of dopaminergic neurons and cognitive impairments, highlighting the role of inflammation in neurodegenerative processes [109,110,111].

However, cytokines like interferon-gamma (IFN-γ) and IL-4 play an essential role in maintaining normal cognitive and social behaviors. Disruptions in the balance of these cytokines during infections can lead to behavioral alterations and neuropsychiatric symptoms [112,113,114].

One of the most important proinflammatory cytokines involved in mediating the inflammatory response within the CNS are TNFα and IL-6 [115,116,117]. TNFα acts through two separate surface receptors (TNF-R 1 and TNF-R 2) and is responsible for the cellular stress response mechanism [115,118]. Central upregulation of TNFα has been linked to dopaminergic neuron death, cognitive dysfunction, memory impairment, and disruption of the BBB, facilitating the entry of leukocytes into the CNS [64,117,119].

In brain homeostasis, IL-6 is present in low concentrations, but during CNS infection elevated IL-6 levels are responsible for memory and cognitive impairment [120,121]. In SARS-CoV-2 infection, IL-6 and TNFα are linked to disease progression and severity, prompting research into treatments targeting these cytokines [122,123]. Some findings suggest that IL-6 can be a crucial biomarker for monitoring and potentially predicting the progression of severe COVID-19 pneumonia [124,125,126]. The effects of key cytokines, including TNFα, IL-1β, IL-6, IFN-γ, and IFN-α, on neurotransmitter pathways and neuronal activity during SARS-CoV-2 infection are summarized in Supplementary Materials—Table S1 [127,128,129,130,131,132,133,134,135,136].

## 4. Inflammatory Response, Cognitive Impairment and Neuropsychiatric Manifestations Associated with COVID-19 Disease

In 2020, the first retrospective analysis was conducted on 214 patients in Wuhan, which found that 36.4% of patients exhibited neurological symptoms [137]. These symptoms were classified into CNS, peripheral nervous system, and skeletal muscle injuries.

Neurological manifestations have been reported in around 80% of patients hospitalized with COVID-19 [138], and patients with severe COVID-19 are at a higher risk of developing neurological complications, although the specific underlying mechanisms remain unclear [16].

Psychiatric disorders such as schizophrenia augmented the risk of COVID-19 infection and mortality, the risk of severe infections increasing during exposure to antipsychotic drugs [139]. Moreover, schizophrenia could be one of the post-COVID-19 sequelae. Thus, Baranova et al. suggested that the risk of schizophrenia was increased by 11% in severe COVID-19 patients [140]. Also, different studies showed that patients with ADHD have an increased risk of infection and of the severe COVID-19 symptoms [141,142].

Although in patients with autism, the long COVID-19 symptoms are difficult to diagnose and manage [143]; aberrant behaviors of autistic patients are related to their mother’s anxiety level. Thus, a worsening of the behavior was correlated with a high level of anxiety in the patients’ mothers [144].

Other retrospective studies reported that one in three COVID-19 survivors were diagnosed with a neuropsychiatric condition within six months of infection, with 13% of them being first-time diagnoses [28,145].

In an observational study of 43 patients infected with SARS-CoV-2, Paterson et al. described the main neurological symptoms of COVID-19 disease: encephalopathy, which is sometimes linked to episodes of delirium and psychosis; CNS inflammatory syndromes such as acute disseminated encephalomyelitis (ADEMs); parainfectious and postinfectious encephalitis; myelitis; ischemic stroke commonly complicated by pulmonary thromboembolic events, and Guillain-Barré syndrome (GBS) [146].

**Figure 4:**
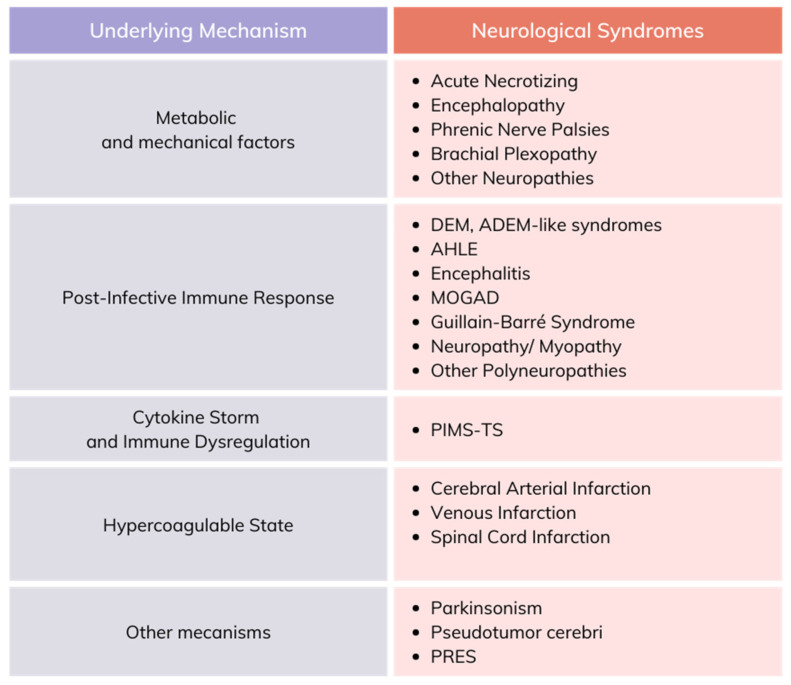
Neurological manifestations of the SARS-CoV infection described in the literature. ADEM—acute disseminated encephalomyelitis; AHLE—acute hemorrhagic leukoencephalitis; MOGAD—myelin oligodendrocyte glycoprotein antibody-associated disease; PIMS-TS—pediatric inflammatory multisystem syndrome; PRES—posterior reversible encephalopathy syndrome.

An Italian study reported that 78% of COVID-19 survivors had cognitive deficits, and 36% experienced depressive symptoms linked to inflammation [26]. The inflammatory response is a common factor in neurodegenerative conditions and mood disorders such as major depressive disorder, where stress can exacerbate inflammation. The most frequent neurological symptoms determined by the SARS-CoV infection described in the literature are represented in Figure 4.

Some studies in the literature reported that COVID-19 patients with neurological disorders show anti-SARS-CoV-2 antibodies in the CSF [23,147,148]. For example, immunoglobulin-G (IgG) antibodies were present in the CSF of all patients with encephalopathy [148], while another study showed a low prevalence of anti-SARS-CoV-2 antibodies in COVID-19 patients [149].

The role of BBB selectivity is to protect the brain from circulating blood cells and to maintain CNS homeostasis. Systemic infection and inflammation can alter the permeability of the barrier, allowing cytokines and inflammatory mediators to enter the CNS, promoting neuroinflammation and neurodegeneration [150].

Some post mortem assays of COVID-19 patients have found undetectable or extremely low concentrations of SARS-CoV-2 RNA in their CSF [63,151,152,153,154,155]. In addition, abnormalities on brain magnetic resonance imaging (MRI), in particular leptomeningeal enhancement, and increased inflammatory markers CSF, are common in COVID-19 patients with neurological symptoms [156]. Jarius et al. found dysfunction of the BBB in 50% of patients with no history of CNS disease [157].

Exposure to the SARS-CoV-2 spike glycoprotein S1 in microglia, mononuclear blood cells, and macrophages triggers the production of proinflammatory cytokines, such as TNFα, IL-8, IL-1β, and IL-6 [158,159,160]. Furthermore, treatment with a TLR4 antagonist in murine macrophages attenuated the proinflammatory cytokine induction and intracellular signaling activation by S1, suggesting that TLR4 signaling appears to play a crucial role in inducing inflammatory responses, including neuroinflammation [159,161,162,163,164]. Other studies suggest that the “S” protein activates the nuclear factor kappa-B (NF-κB) pathway via TLR2 in a MyD88-dependent manner. This pathway plays a crucial role in the cytokine storm observed during COVID-19 [165,166].

## 5. Acute Neuropsychiatric Complications of COVID-19

In terms of severe acute COVID-19 complications, these comprise ischemic or hemorrhagic stroke, hypoxic-anoxic injuries, PRES, and acute disseminated myelitis, as well as the occurrence of neuromuscular disorders, such as GBS, which can lead to persistent or even permanent neurological impairment [138]. A study examining the medical records of more than 40,000 COVID-19 patients revealed that 22.5% had neurological and/or psychiatric complaints, of which anxiety and related disorders were the most common [167].

There are also reports in the literature of psychotic episodes in patients during the acute phase of COVID-19. One study found that the most common neuropsychiatric syndrome was early psychosis, followed by other associated psychiatric disorders [168,169]. In a Spanish cohort of COVID-19 inpatients, approximately 20% presented neuropsychiatric symptoms, including insomnia, anxiety, depression, and psychosis [17]. Case reports have also documented the presence of manic and psychotic symptoms in patients with COVID-19 who had no prior psychiatric diagnosis [170,171].

The most frequently reported acute symptoms of COVID-19 in the literature, together with the possible associated pathophysiology, are presented in Table 1.

## 6. Chronic Neuropsychiatric Symptoms and Post-Recovery

There are several neurological and psychiatric diseases known to be mediated by a neuroinflammatory process, like the one described for COVID-19. The molecular mechanisms causing this inflammation may have a considerable impact on the development and progression of neurodegenerative diseases, but also of psychiatric disorders, especially mood disorders, as their pathogenesis also involves neuroinflammatory mechanisms. Research to date has associated increased levels of C-reactive protein with the incidence of cognitive deficits post-infection in patients who have recovered. This would suggest a possible role of inflammation in the cognitive deficit reported by this group of post-recovery patients [195]. Maamar et al. found that patients who had elevated serum levels of inflammatory markers, such as C-reactive protein, neutrophil-to-lymphocyte ratio, neutrophils, and fibrinogen exhibited prolonged COVID-19 symptoms [196].

According to the National Academies of Sciences, Engineering, and Medicine in 2024, long COVID-19, also known as post-acute sequelae of SARS-CoV-2 infection, is defined broadly as signs, symptoms, and conditions that continue or develop after the initial phase of COVID-19 infection. These manifestations persist for four weeks or more, can be multisystemic, and may exhibit a relapsing-remitting pattern with potential for progression or worsening over time. This term encompasses various health issues that might have different biological causes and risk factors and unfortunately, there are currently no consensus-based diagnostic criteria for long COVID-19 [197].

Although evidence for a causal link between COVID-19-associated neuroinflammation and the onset of psychiatric disorders remains limited, it is nevertheless possible that this category of patients with neuroinflammatory impairment may be more likely to develop depression, anxiety, and long-term PTSD [198,199,200].

One can also hypothesize about the long-term CNS effects of COVID-19 based on the physiopathological mechanisms implicated in the development of long-term neuropsychiatric disorders associated with SARS-CoV-1 and Middle East respiratory syndrome (MERS). A study of SARS-CoV-1 survivors showed that 55% of them experienced PTSD, 39% developed depression, over 32% experienced panic disorder, and 15.6% exhibited an obsessive-compulsive disorder [201].

Mood disorders, mainly depression, are also more common in post-infection COVID-19 patients than in those recovering from influenza or other respiratory tract infections [28].

In patients with long COVID-19 syndrome, including those who have not been hospitalized, at least one persistent neuropsychiatric symptom has been reported [202].

In 2022, about 6.9% of adults and 1.3% of children in the U.S. experienced long COVID-19 at some point. By January 2023, the prevalence among U.S. adults was recorded at 5.9%, which increased to 6.8% by January 2024, demonstrating a continuing and significant burden of the disease. Although the overall prevalence has decreased since mid-2022, long COVID-19 still presents a substantial health burden. Approximately 22% of adults with long COVID-19 reported significant activity limitations as of January 2024 [197]. The most frequent long COVID-19 symptoms reported in the literature are presented in Table 2 [197].

A retrospective cohort study was conducted in 1,284,437 COVID-19 patients over two years. The study found that after six months, most patients still faced a significantly increased risk of neurological damage. Additionally, there were but a few conditions, such as encephalitis, GBS, nerve and plexus damage, and Parkinson’s disease that did not show an increased incidence among COVID-19 patients. At the same time, the incidence of cognitive deficits, dementia, psychiatric disorders, epilepsy, and seizures was still high even after two years. Children were at greater risk of developing cognitive deficiencies, insomnia, intracranial hemorrhage, ischemic stroke, nervous disorders, epilepsy, or seizures, while adults were more likely to develop common psychiatric disorders [203]. As multiple studies have shown, the symptoms of COVID-19 infection in children are less severe than in the adult population. Also, older age is associated with an increased risk of long-term symptoms [204]. Another characteristic of the pediatric population is that patients with multisystem inflammatory syndrome (MIS) have a higher prevalence of long COVID, including neurologic and psychiatric symptoms, compared to patients without MIS [205]. Over a longer follow-up period, neurological and psychiatric symptoms due to long COVID were observed to decrease. Symptoms due to long COVID in children seem to be reversible, even though in some cases it may take a long time [205].

## 7. Diagnosis of COVID-19 Neuropsychiatric Manifestations and Biomarkers Used to Monitor Neurological and Psychiatric Alterations in COVID-19 Patients

Clinical assessment is an important step in diagnosing neuropsychiatric symptoms in patients with COVID-19 disease. This involves a series of laboratory tests needed to rule out other diagnoses such as metabolic disorders, CNS infections, and psychiatric disorders.

If the symptoms are consistent with focal neurological deficits, or if there is a suspicion that they may be associated with COVID-19 disease, a brain MRI is strongly recommended.

As for the diagnostic criteria for mood disorders such as major depressive disorder and anxiety disorders in COVID-19 patients, they do not differ from the standard criteria used in their diagnosis.

### 7.1. Biomarkers

Monitoring neurological and psychiatric changes in patients with COVID-19 requires a complex approach that includes clinical assessment, neuroimaging, and evaluation of specific biomarkers associated with CNS dysfunction and psychological distress. Biomarkers play an important role in establishing the diagnosis, but also in predicting disease severity.

Multiple studies have examined serum biomarkers in order to evaluate the nature of CNS injury. Plasma NfL (pNfL), an intra-axial structural protein, is a validated biomarker for detecting neuro-axial injury, while plasma GFAP (pGFAP) is an astrocytic cytoskeletal protein that is overexpressed in activated astrocytes [206,207,208,209]. Both markers, including also other CSF biomarkers and ubiquitin C-terminal hydrolase L1 (UCH-L1), have been shown to be elevated in the acute phase of SARS-CoV-2 infection, suggesting that CNS damage is linked to neuronal impairment and astrocyte activation during acute infection [105,210,211,212,213,214].

Furthermore, additional studies in the literature support the relation between GFAP levels and the development of severe disease in COVID-19 patients. GFAP is a glial cytoskeletal protein mainly expressed in astrocytes, which regulates the morphology and function of these cells in the CNS [215,216,217]. Serum levels of GFAP in healthy patients are very low, but in case of neuronal injury, GFAP levels increase [99,216]. To date, there are no specific biomarkers for the SARS-CoV-2 infection. However, several biomarkers are commonly used to assess the severity of COVID-19, the inflammatory response and potential complications. The most common non-specific biomarkers, used to monitor neurological and psychiatric impairment in COVID-19 disease and that are described in the literature, are listed in Table 3.

### 7.2. Imagistic Investigations

Together with specific biomarkers, neuroimaging plays an important role in observing microstructural changes with implications for cognitive function and neuro-psychiatric outcomes. Thus, in COVID-19 patients with neurological manifestations, neuroimaging could detect underlying causal pathology.

Neuroimaging helps specialists to observe the microstructural changes in cognitive function and neurological outcomes in COVID-19 patients. Thus, different neuroimaging techniques dominated by MRI, computed tomography (CT), and positron emission tomography (PET) could be used in evaluating the abnormalities attributable to hypoxic, vascular, and inflammatory pathology. Advanced neuroimaging revealed cerebral abnormalities of olfactory system, cortical hypoperfusion, BBB leakage, white matter microstructural integrity alterations, altered glucose metabolism in the COVID-19 brain, acute ischemic stroke, cerebral venous thrombosis, and meningoencephalitis. [30,218,219,220].

### 7.3. Psychological Tools

Different tools were used for the evaluation of the mental status of the patients with COVID-19 such as:

- COVID-19 Stress Scale for understanding the distress associated with COVID-19 and for identifying people in need of mental health services. Based on 36 items, the scale identifies 5 factors: (1) danger, (2) fears about economic consequences, (3) xenophobia, (4) compulsive checking and reassurance seeking, and (5) traumatic stress symptoms about COVID-19.
- Depression, Stress and Anxiety Scale 21, a self-report questionnaire based on 21 questions,
- Generalized Anxiety Disorder-7 questionnaire for evaluation of the generalized anxiety symptoms during the 2 weeks (based on the Likert Scale),
- The COVID-19 Peritraumatic Distress Index contains 24 items regarding the anxiety, depression, specific phobias, cognitive change, avoidant and compulsive behaviors, physical symptoms and loss of social functioning in the past week (based on the Likert Scale) [221,222].

## 8. Pharmacotherapy of Neuropsychiatric Disorders and Management Strategies Associated with COVID-19

The primary treatment for most neuropsychiatric events associated with COVID-19 is supportive therapy. A multidisciplinary approach is essential to effectively manage the comorbidities of COVID-19 patients.

A retrospective UK study of 184,986 adult hospitalized patients revealed that patients cotreated with dexamethasone and remdesivir experienced fewer neurological events such as strokes, seizures, encephalitis, or meningitis compared with those receiving standard treatment [223].

Also, in patients with severe COVID-19 who received either dexamethasone or remdesivir or their combination, a reduction in neurological manifestations was observed.

The effects of other drugs on reducing neurological impairment, such as tocilizumab, ritonavir, baricitinib, or nirmatrelvir, which have been effective in treating SARS-CoV-2 infection, have not yet been reported. However, these drugs in turn target inflammation or viral replication and are expected to have a similar effect on neurological complications. Likewise, seizures were reduced in COVID-19 patients with severe disease who were administered either dexamethasone or remdesivir or a combination of the two. Incidence of meningitis and/or encephalitis events also decreased in the severe disease groups following dexamethasone or combination therapy, which suggests the possible benefit of reducing CNS inflammatory events.

Therefore, the management of neurological and psychiatric manifestations of COVID-19 involves a tailored approach based on specific symptoms. In patients presenting with acute neurological complications, such as encephalitis or stroke, antiepileptic drugs, antithrombotic therapy, and supportive therapy, respectively, may be indicated depending on the clinical presentation. Psychiatric symptoms, including anxiety, depression, and psychosis, may require initiation of serotonin-norepinephrine reuptake inhibitors (SNRIs), selective serotonin reuptake inhibitors (SSRIs), or antipsychotic drugs. However, particular attention should be paid to potential drug interactions and adverse effects, especially in patients with underlying medical conditions or who use concomitant medication [224].

However, more research is needed to corroborate this. Immunization programs have been successful in significantly decreasing the incidence and severity of COVID-19 cases. Several clinical studies performed in post-vaccination COVID-19 infections concluded that the use of the vaccine is associated with a lower risk of hypercoagulopathy or venous thromboembolism, seizures, or psychotic disorder especially after the second vaccine dose [225]. Also, a reduction in the risk of neuropsychiatric disorders involved in post-vaccinated COVID-19 patients was reported [226]. Moreover, the key finding of a recent study published in June 2024, was that the median self-reported time to recovery from SARS-CoV-2 infection was approximately 20 days, 1 in 5 COVID-19 patients had not fully recovered from the infection after 90 days, and interestingly, vaccination before the infection and infection with an Omicron variant was associated with shorter recovery times [227].

While adenovirus vaccines have been associated with a small number of neurological disorders, such as GBS, studies suggest that the risk of neurological events caused by SARS-CoV-2 infection greatly exceeds the risk following vaccination [228]. Evidence from a recent meta-analysis suggests that vaccination lowers the risk of developing post-COVID-19 disease, with patients who received two vaccine doses showing a significantly lower risk compared to those who remained unvaccinated. Nevertheless, further investigations are needed in order to improve the treatment of comorbidities associated with a severe form of COVID-19 disease and thus to reduce the risk of neurological complications [229].

**Figure 5:**
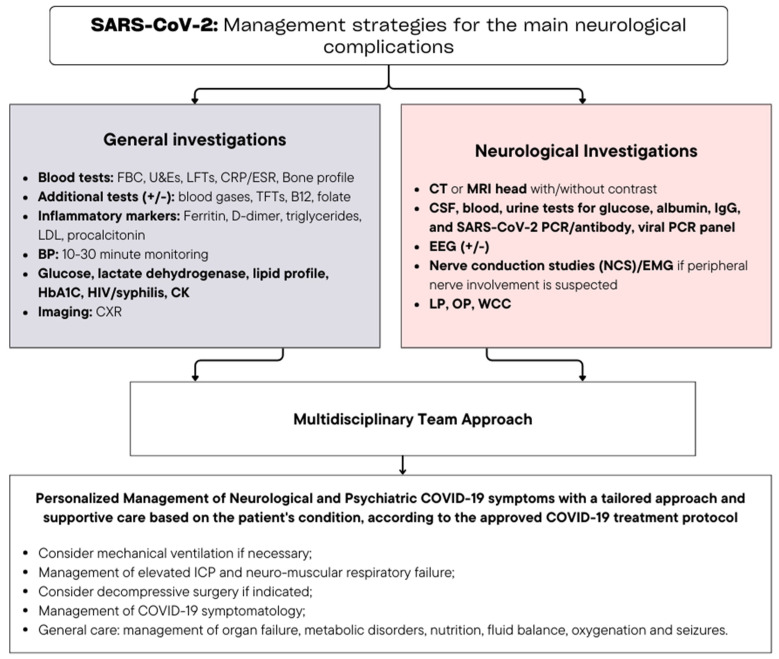
Management and diagnostic approaches for neurological sequelae in SARS-CoV-2 infection [224].

Figure 5 describes the management and diagnostic approaches for the key neurological sequelae in SARS-CoV-2 infection (Supplementary Materials—Table S2) [230].

## 9. Conclusions

The SARS-CoV-2 virus has a profound impact beyond its primary respiratory manifestations, significantly affecting the central and peripheral nervous systems. This review highlights the wide range of neurological and psychiatric symptoms associated with COVID-19, from mild conditions such as headache and anosmia to severe complications such as stroke and encephalopathy. Long-term neuropsychiatric disorders, including cognitive dysfunction, anxiety, and depression, highlight the long-term consequences of SARS-CoV-2 infection.

Understanding the underlying mechanisms of these neuropsychiatric manifestations is critical. Key factors include the ability of the virus to invade the CNS, the resulting inflammatory response and the role of ACE2 receptors in neuroinflammation. The review also highlights the importance of specific biomarkers in diagnosing and monitoring CNS involvement, which can guide treatment strategies.

Effective management of the neurological and psychiatric effects of COVID-19 requires a multidisciplinary approach combining supportive care with targeted pharmacological treatments. Further research into these mechanisms and management strategies is essential to improve patient outcomes and address the long-term mental health effects of COVID-19. Neuropsyciatric implications must be considered when developing health strategies. Not at least, further studies would be helpful to fill the gap regarding the developing of the neuropsychiatric symptoms in different populations, or regarding the neuropsychiatric outcomes in vaccinated or non-vaccinated population compared to other viral infections.

## Figures and Tables

**Table 1:** Most common acute symptoms of COVID-19 disease.

Acute Symptoms of COVID-19 Disease	Possible Associated Pathophysiology
**Anosmia, Ageusia**	Nasal congestion, resulting in the loss of fine olfactory receptor cell endings, making them unable to detect odors [172,173,174]; SARS-CoV-2 virus predominantly colonizes the patients’ nasal cavity, triggering inflammation in the olfactory nerves and causing structural damage to the receptors [173,175,176]; High expression of ACE2 receptors in the tongue cells as compared to other mouth tissues makes them more susceptible to viral binding [177,178,179].
**Neurogenic respiratory failure**	SARS-CoV-2 can infect nerve cells in the myelencephalon, which is responsible for regulating several basic functions of the autonomic nervous system, including respiration, cardiac function and vasodilation [180]; Involved secondary mechanism due to increased inflammatory markers like TNFα and IL-8, which have been associated with pleocytosis [181].
**GBS**	GBS may be caused by the neuroinvasion of SARS-CoV-2, causing the side effect of demyelination [18,182,183,184]; The viral infection may trigger an exaggerated immune response via molecular mimicry, which leads to the demyelination of peripheral nerves [185]; First GBS case in a COVID-19 patient reported in January 2020 [186].
**Encephalopathy**	The most common neurological complication seen in COVID-19 patients from Intensive C [18,187]; Symptoms: restlessness, confusion, delirium [188,189,190]; Can occur in less severe cases, affecting young adults [191]; The first case of acute necrotizing hemorrhagic encephalopathy associated with COVID-19 was reported in 2020 [192]; The base mechanism involves the onset of cytokine storm in the brain, leading to BBB dysfunction [193]; Cases of toxic metabolic encephalopathy were also reported in the literature [191], with risk factors including age, male gender, diabetes, pre-existing conditions [194].

**Table 2:** Common long COVID-19 symptoms reported in the literature according to the consensus study report 2024 of the National Academies of Sciences, Engineering, and Medicine [197].

Category	Long COVID-19 Symptoms
**Neuropsychiatric**	Cognitive dysfunction (brain fog); Headaches; Dizziness; Concentration difficulties; Memory impairments; Mood changes; Tinnitus; Visual disturbances; Chronic fatigue syndrome; Parosmia; Anosmia; Sleep disorders; Anxiety; Depression; Fibromyalgia.
**Respiratory**	Shortness of breath; Chronic cough; Pulmonary complications; Breathlessness.
**Cardiovascular**	Chest pain; Palpitations; Tachycardia; Endothelial dysfunction; Coagulation disorders.
**Gastrointestinal**	Nausea; Diarrhea; Loss of appetite; Weight loss; Gastroesophageal reflux disease; Irritable bowel syndrome; Gut dysbiosis.
**Musculoskeletal**	Muscle weakness; Joint pain; Musculoskeletal pain.
**Other**	Post-exertional malaise; Rash; Sore throat; Hair loss; Pins and needles sensation; Painful lymph nodes; Swelling in extremities; Reduced exercise tolerance; Bladder control issues; Sexual dysfunction; Persistent hiccups; Endocrine dysfunction; Kidney dysfunction; Immune dysregulation; Mast Cell Activation Syndrome.

**Table 3:** Non-specific biomarkers used to monitor neurological and psychiatric impairments in COVID-19 disease.

Biomarker	Description
**Neurological Impairment**	
**NfL**	Neuronal cytoskeletal protein released into the CSF and blood following neuronal injury or degeneration; Elevated in COVID-19 patients with neurological complications; Indicates CNS involvement and progression.
**S100B protein**	Calcium-binding protein expressed predominantly in astrocytes and oligodendrocytes; Increased levels indicate glial activation and neuroinflammation; Reflects severity of neurological complications in COVID-19, including BBB dysfunction.
**Other CSF biomarkers**	Includes protein levels, cell counts, and inflammatory markers like IL-6, IL-8, TNF-α; Diagnosis and characterization of neuroinflammatory disorders in COVID-19.
**UCH-L1**	UCH-L1 levels were higher in patients needing ICU transfer; UCH-L1 linked to neuronal injury.
**Psychiatric Impairment**	
**Brain-derived neurotrophic factor (BDFN)**	Neurotrophin involved in neuronal survival, synaptic plasticity and mood regulation; Low levels associated with psychiatric symptoms in COVID-19.
**Peripheral cytokine profiles**	Elevated cytokine levels, including IL-6, TNFα, and IFN-γ were associated with mood disorders, cognitive impairment, and psychosis in COVID-19 patients.

## Data Availability

Data contained within the article.

## Supplementary Materials

The following supporting information can be downloaded at: https://www.mdpi.com/article/10.3390/v16121811/s1, Table S1: The effects of cytokines on neurotransmitter pathways and neuronal activity; Table S2: Therapeutic management of COVID-19 disease, according to NICE guidelines, version May 2024.

## Abbreviations

ACE2	angiotensin-converting enzyme 2
ADEM	acute disseminated encephalomyelitis
AHLE	acute hemorrhagic leukoencephalitis
AKI	acute kidney injury
BBB	blood-brain barrier
BDNF	brain-derived neurotrophic factor
BP	blood pressure
B12	vitamin B12
C1q	complement component 1q
CNS	central nervous system
COVID-19	Coronavirus Disease 2019
CRP	C-reactive protein
CSF	cerebrospinal fluid
CT	computed tomography
CXR	chest X-Ray
EEG	electroencephalogram
EMG	electromyography
eNOS	endothelial nitric oxide synthetase
ESR	erythrocyte sedimentation rate
FBC	full blood count
GBS	Guillain-Barré syndrome
GFAP	glial fibrillary acidic protein
HbA1C	hemoglobin A1c
IgG	immunoglobulin G
ICU	intensive care unit
IFN-γ	interferon-gamma
IL	interleukin
LFTs	liver function tests
MERS	Middle East Respiratory syndrome
MIS	multisystem inflammatory syndrome
MOGAD	myelin oligodendrocyte glycoprotein antibody-associated disease
MRI	magnetic resonance imaging
NCS	nerve conduction studies
NfL	neurofilament light chain
NICE	National Institute for Health and Care Excellence
NO	nitric oxide
OP	opening pressure
PCR	polymerase chain reaction
pGFAP	plasma glial fibrillary acidic protein
PIMS-TS	pediatric inflammatory multisystem syndrome
pNfL	plasma neurofilament light chain
PRES	posterior reversible encephalopathy syndrome
PTSD	post-traumatic stress disorder
RAAS	renin-angiotensin-aldosterone system
RNA	ribonucleic acid
SARS	severe acute respiratory syndrome
SARS-CoV-1	severe acute respiratory syndrome coronavirus 1
SARS-CoV-2	severe acute respiratory syndrome coronavirus 2
SNRI	serotonin-norepinephrine reuptake inhibitors
SSRI	selective serotonin reuptake inhibitors
TFTs	thyroid function tests
TLR	toll-like receptor
TNFα	tumor necrosis factor-alpha
UCH-L1	Ubiquitin C-Terminal Hydrolase L1
U&Es	urea and electrolytes
WCC	white cell count
WHO	World Health Organization
