# Supplementary material for: Neuropsychiatric Burden of SARS-CoV-2: A Review of Its Physiopathology, Underlying Mechanisms, and Management Strategies
Source: Viruses. 2024 Nov 21;16(12):1811. doi: 10.3390/v16121811 (PMC11680421; doi:10.3390/v16121811)
Supplement: Supplementary file 1 [file viruses-16-01811-s001.zip › viruses-3314800-supplementary.pdf]

Table S1. The effects of cytokines on neurotransmitter pathways and neuronal activity.

Cytokine	Effect
**TNF**	Induction of dopaminergic neuron death by microRNA targeting mitochondrial complex-1 [117];<br>Inhibition of morphine-triggered activation of dopaminergic neurons in the ventral tegmental area [107,125];<br>Cognitive and motor impairment [115,126,127];<br>Enhancing TNF-R2 and inhibiting TNF-R1 adult neurogenesis [116];<br>Cognitive impairment through a mechanism linked to TNF-R1 signaling in astrocytes [128];
**IFN-γ**	Chemokine ligand 9-mediated dopaminergic neuronal death [129];<br>Induction of long-term potentiation depression mediated by chemokine ligand 10 [130];<br>Improvement of adult neurogenesis [131];<br>Dysfunction of the cerebellum [132].
**IL-1β**	Blockage of cerebellum function [133];<br>Dopaminergic degeneration with age-related motor decline [134].
**IFN-α**	Activation of the kynurenine pathway, resulting in decreased production of the neurotransmitters serotonin and dopamine [135];<br>Excitotoxicity or reduced glutamatergic signalling due to interaction with N-Methyl-D-aspartic acid (NMDA) receptors [135];<br>Inhibition of synaptic plasticity and adult neurogenesis[136].

Table S2. Therapeutic management of COVID-19 disease, according to NICE guidelines, version May 2024 [230].

Therapeutic representatives	NICE guidelines recommendation, version May 2024
	**Antiviral drugs**
**Nirmatrelvir and Ritonavir**	Adults, only if they do not require supplemental oxygen for COVID-19, and have any of the following:<br>An increased risk of progression to severe COVID-19;<br>Age ≥ 70 years;<br>BMI ≥ 35 kg/m²;<br>Diabetes;<br>Heart failure.
**Ritonavir**	Hospitalized adults and pediatric patients ≥ 40 kg who are at increased risk of severe COVID-19;<br>Hospitalized pediatric patients, only if they are aged 4 weeks to 17 years and weigh at least 3 kg, have pneumonia, and need supplemental oxygen.
**Molnupiravir**	5-day treatment for COVID-19 in adults who do not require supplemental oxygen, are within 5 days of symptom onset, and are at high risk of disease progression.
**Sotrovimab**	Treatment for COVID-19 in adults and pediatric patients aged 12 and over ≥ 40 kg, who do not require supplemental oxygen, are at increased risk of progression to severe COVID-19 disease, and the combination of Nirmatrelvir plus Ritonavir is contraindicated.
	**Corticosteroids**
**Dexamethasone**	COVID-19 patients who require oxygen supplementation to achieve prescribed oxygen saturation levels, or who require supplemental oxygen but cannot tolerate it;<br>If dexamethasone is not available or cannot be used, hydrocortisone or prednisolone can be used instead;<br>Treatment with corticosteroids is recommended for 10 days unless there is a clear indication to stop earlier;<br>Posology: 6 mg orally or intravenously for 10 days. The intravenous formulation should only be considered if oral tablets or solutions are inappropriate or unavailable;<br>Dosage for children with a greater than 44-week corrected gestational age: 150 micrograms/kg orally, nasogastrically, or intravenously once a day for 10 days (maximum 6 mg);<br>The use of corticosteroids is not recommended in COVID-19 patients who do not require oxygen supplementation.
**Prednisolone**	Alternative corticosteroid if dexamethasone cannot be used;<br>Posology: 40 mg orally once daily for 10 days;<br>Dosage for children with a greater than 44-week corrected gestational age: 1 mg/kg orally, nasogastrically, or intravenously once a day for 10 days (maximum 40 mg).
**Hydrocortisone**	Alternative corticosteroid if dexamethasone cannot be used;<br>Posology: 50 mg intravenously every 8 hours for 10 days; this may be continued for up to 28 days for people with septic shock.
	**SARS-CoV-2 Specific Monoclonal Antibodies**
**Casirivimab and Imdevimab**	• First emergency use authorization for the treatment of COVID-19 in November 2020 in the USA, followed by the EMEA authorization in February 2021;<br>On January 24, 2022, the FDA withdrew the emergency use authorization (EUA) for casirivimab and imdevimab;<br>The combination is not an approved indication for the treatment of COVID-19 anymore.
	**Interleukin-6 Receptor Antagonist**
**Tocilizumab**	Treatment option for COVID-19 disease in adults who require supplemental oxygen or mechanical ventilation and are already receiving systemic corticosteroid therapy;<br>The use of tocilizumab should only be considered after the exclusion of other bacterial or viral infections (other than SARS-CoV-2) that may be exacerbated by tocilizumab;

	The efficacy of tocilizumab in treating COVID-19 in patients without elevated C-reactive protein levels has not been established.
**Janus Kinase (JAK) Inhibitor**	
**Janus Kinase (JAK) Inhibitor**	Hospitalised patients over 2 years of age who require supplemental oxygen, are receiving corticosteroid therapy, and who have been excluded from other bacterial or viral infections (other than SARS-CoV-2) that may be exacerbated by baricitinib.<br>Starting March 2023, the use of baricitinib for COVID-19 patients is considered off-label.
**Antibiotics**	
**Azithromycin, Doxycycline, or any other type of antibiotic**	The use of antibiotics for the prevention or treatment of COVID-19 is not recommended, unless there is a clinical suspicion of an additional bacterial co-infection.
**Inhaled Corticosteroids**	
**Budesonide**	Budesonide should only be used for the treatment of COVID-19 as part of a clinical trial.
**Anti-Mitotic Drugs**	
**Colchicine**	The use of Colchicine for the prevention or treatment of COVID-19 is not recommended.
**Anti-Parasitic Agents**	
**Ivermectin**	The use of Ivermectin for the treatment of COVID-19 is not recommended.
**Monoclonal Antibodies**	
**Tixagevimab + Cilgavimab**	The combination is not recommended, as per their marketing authorization, for the treatment of COVID-19 in adults who do not require supplemental oxygen and are at an increased risk of developing severe COVID-19;<br>EMA authorization: Used to prevent COVID-19 in adults and adolescents (≥12 years and ≥40 kg);<br>As of January 26, 2023, the FDA has withdrawn the EUA of this combination.
**Vitamins**	
**Vitamin D**	The use of Vitamin D for the treatment of COVID-19 is not recommended.
